# Association between diabetes and heart failure after coronary artery bypass grafting: Danish register-based cohort study

**DOI:** 10.1007/s00392-025-02594-8

**Published:** 2025-02-24

**Authors:** Benedicte Bay Oxholm Brodersen, Line Tribler Kristiansen, Sidsel le Fevre Karlsen, Jeppe Hauch, Jan Jesper Andreasen, Kristian H. Kragholm, Maria Lukács Krogager, Lars Valeur Køber, Peter Christian Leutscher, Dorte Melgaard, Nisha I. Parikh, Morten Schou, Peter Søgaard, Christian Torp-Pedersen, Marc Meller Søndergaard

**Affiliations:** 1 The Faculty of Medicine, Aalborg University, Aalborg, Denmark; 2 Department of Cardiothoracic Surgery, Aalborg University Hospital, Aalborg, Denmark; 3 Department of Clinical Medicine, Aalborg University, Aalborg, Denmark; 4 Department of Cardiology, Aalborg University Hospital, Aalborg, Denmark; 5 Unit of Clinical Biostatistics and Epidemiology, Aalborg University Hospital, Aalborg, Denmark; 6 Department of Cardiology, Rigshospitalet, Copenhagen University Hospital, Copenhagen, Denmark; 7 Center for Clinical Research, North Denmark Regional Hospital, Hjørring, Denmark; 8 Department of Clinical Medicine, Copenhagen University, Copenhagen, Denmark; 9 North Denmark Regional Hospital, Hjørring, Denmark; 10 Department of Medicine, Division of Cardiology, University of California San Francisco, San Francisco, CA USA; 11 Department of Cardiology, Herlev and Gentofte Hospital, Copenhagen, Denmark; 12 Department of Cardiology, Nordsjællands Hospital, Copenhagen, Denmark

**Keywords:** Heart failure, Diabetes mellitus, Coronary artery bypass grafting, Mortality

## Abstract

**Background:**

Ischemic heart disease (IHD) is the leading cause of mortality in the world with an increasing incidence. One of the interventions to treat IHD is coronary artery bypass grafting (CABG) and people with diabetes mellitus (DM) account for approximately one quarter of all patients who undergo coronary revascularization. Furthermore, people with DM have a higher risk of mortality due to heart failure (HF).

**Objective:**

We aim to describe the risk of developing HF after CABG in patients with versus without DM.

**Methods:**

Through a large nationwide register-based cohort study, patients who underwent CABG from January 1, 2000 to December 31, 2020 were included. In addition to Cox regression, g-formula methods based on multivariable Cox regression were performed to estimate the absolute risk (AR) and risk difference (RD) of the association between DM status and HF outcome, and between DM status and mortality.

**Results:**

A total of 34,855 patients were included in this study, consisting of 6909 (19.8%) DM patients. The AR of HF after CABG in the 10th year was 35.1% versus 26.4% for patients with versus without DM (*p* < 0.001), respectively. The RD of HF for each exceeding year (3.7 percentage point (pp.) in the 1st year versus 8.6 pp. in the 10th year) was higher for patients with DM compared to those without DM.

**Conclusion:**

The risk of HF was significantly higher up to ten years after CABG in patients with DM compared to those without DM.

**Graphical abstract:**

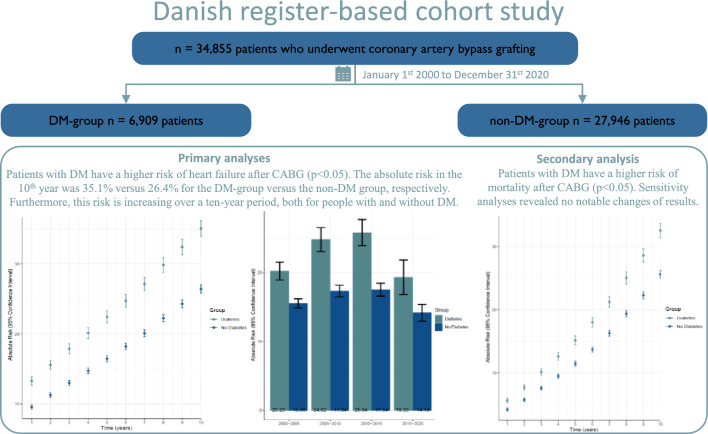

**Supplementary Information:**

The online version contains supplementary material available at 10.1007/s00392-025-02594-8.

## Introduction

Diabetes mellitus (DM) is associated with an increased risk of ischemic heart disease (IHD) including acute myocardial infarction (AMI) leading to increased morbidity and mortality [1–4]. People with DM account for approximately one quarter of all patients who undergo coronary artery revascularization, such as percutaneous coronary intervention (PCI) or coronary artery bypass grafting (CABG) [5–9]. Although PCI often is the primary intervention, CABG is still the preferred procedure to treat complex IHD, especially in patients with DM with three-vessel disease [10].

Following CABG, HF is one of the most common causes of death, as injury to myocardial tissue is likely during cardiac surgery represented as arrhythmia, myocardial stunning, low cardiac output, and perioperative myocardial infarction [9, 11–14]. Generally, people with DM have a four- and eight-fold risk of mortality in men and women, respectively, compared to people without DM, especially mortality caused by HF which is two- to four-fold in DM patients [5, 15, 16]. Furthermore, the onset of HF is on average ten years earlier in people with DM, compared to people without DM, where one of the causes might be diabetic cardiomyopathy [3, 5].

Extensive research has gone into addressing risk factors of poor outcome after CABG. However, the risk of developing HF after CABG for people with DM is still unknown [6, 11, 17, 18]. Thus, we aimed to describe the risk of developing HF after CABG among Danish patients with DM compared to patients without DM. We hypothesized that patients with DM have a higher risk of developing HF after CABG.

## Methods

### Study setting and population

A nationwide, register-based, cohort study of Danish patients with a history of first-time, isolated CABG between January 1, 2000 and December 31, 2020 was performed. 

Patients who underwent concomitant valve surgery repair were excluded. Furthermore, we excluded patients diagnosed with HF prior to CABG. Patients who emigrated and/or immigrated before inclusion were excluded to limit loss to follow-up. For a full list of procedure codes used to define CABG, please refer to Online Resource Table 1 [19].

### Data sources

The study population was identified from the Danish National Patient Registry, as all individuals with permanent residence in Denmark have a unique Central Personal Registration number, making health data accessible to match across registries.

Date of birth, sex, emigration, and immigration were obtained from The Danish Civil Registration System, data on education were obtained from the Danish Education Registers, and date of death was obtained from the Danish National Registry of Causes of Death [20–22].

Data on prescription drugs were obtained from The Danish National Prescription Registry where data on heart failure, the various comorbidities together with the date of diagnosis, were obtained from the Danish National Patient Registry [23, 24]. These two registries contain information about date of dispensing, Anatomical Therapeutic Chemical (ATC) drug classification code, and information on all hospital admissions in Denmark [23, 24]. National laboratory data were obtained. However, this data source has reduced availability of laboratory data during the study period, starting from 2010 onward and with increasing coverage over time.

### Exposure

DM was the primary exposure. All patients who received an anti-diabetic drug (ATC code A10) and/or a hospital admission, outpatient contact, or an emergency room contact linked with DM (ICD-9 code 250 and ICD-10 code DE10, DE11, DE12, DE13, DE14), as done previously [19], five years prior to CABG were included in the DM group.

### Outcome

The primary outcome was HF and was defined as either a hospital admission, outpatient contact, or an emergency room contact with an HF diagnosis. HF was defined in accordance with ICD-10 codes as done previously [19]. For specificity, sensitivity, and ICD-10 codes, please refer to Online Resource Table 1 [25].

The secondary outcome was mortality, obtained from the Danish National Registry of Causes of Death as mentioned earlier [21].

### Covariates

Included were the following comorbidities five years prior to CABG according to ICD-10 and ATC codes: hypertension, chronic kidney disease (CKD), chronic obstructive pulmonary disease, atrial fibrillation, and cancer (excluding non-melanoma skin cancer). Hypertension was defined as done previously [19, 26]. Baseline medication was defined as dispensed prescription five years prior to inclusion and consisted of acetylsalicylic acid, antiplatelet medication (clopidogrel, prasugrel, and ticagrelor), anticoagulant medication (non-vitamin K antagonist oral anticoagulant: rivaroxaban, apixaban, dabigatranetexilat, and edoxaban, along with vitamin K antagonist: warfarin and phenprocoumon), thyroid medication, lipid modifying agents, ACE inhibitors, diuretics, and beta blockers [19]. Education level was included and divided into four groups as done previously: (0) basic school, (1) high school, (2) medium education, and (3) high education [27]. Baseline estimated glomerular filtration rate (eGFR), hbA1c, blood glucose, LDL cholesterol, and creatinine were reported. For a complete list of Nomenclature, Properties and Units (NPU)- ICD-10, ATC codes used, please refer to Online Resource Table 1.

### Statistical analyses

Categorical variables are presented using counts and percentages, and continuous values are summarized by means and standard deviations. Differences in continuous variables were evaluated using *t* tests, and *χ*^2^ tests were used to evaluate differences in the categorical variables. In addition to a comparison between patients with DM and patients without, baseline characteristics were reported in time intervals: years 2000–2005, 2005–2010, 2010–2015, and 2015–2020. Additionally, the use of anti-diabetic medication, among patients with diabetes, at baseline was reported, according to the same time intervals.

Two multivariable Cox proportional hazards regression were performed to estimate absolute risks, the first one to assess the association between DM and HF and the second one to assess the association between DM and all-cause death. Models were adjusted for sex, age, atrial fibrillation, cancer, chronic obstructive pulmonary disease, CKD, hypertension, lipid modifying agents, and thyroid medication. By using g-formula methods based on multivariable Cox regression, standardized absolute risk (AR) and risk difference (RD) in yearly intervals for a ten-year period were derived [28]. Patients were followed from the date of CABG until either HF, emigration, death, or December 31, 2020. Together with aforementioned models, a stratified analysis based on four different time intervals, years 2000–2005, 2005–2010, 2010–2015, and 2015–2020, was conducted.

Six different sensitivity analyses were performed: one with AMI as endpoint, one with AMI and PCI as secondary endpoint, one adjusted for education level, one excluding patients who had HF within 30 days postoperatively, and two analyses based on sex [27].

Data management and analyses were performed using Rstudio (package 3.5.3, version 1.0.143, Rstudio, Inc., Boston, MA). A *p* value < 0.05 was considered statistically significant.

### Ethical approval

In Denmark, ethical approval is not required for register-based studies. The data usage was authorized by the appropriate data responsible institution, the Capital Region of Denmark, in accordance with General Data Protection Regulation (approval number P-2019-348).

## Results

### Study population

**Fig. 1:**
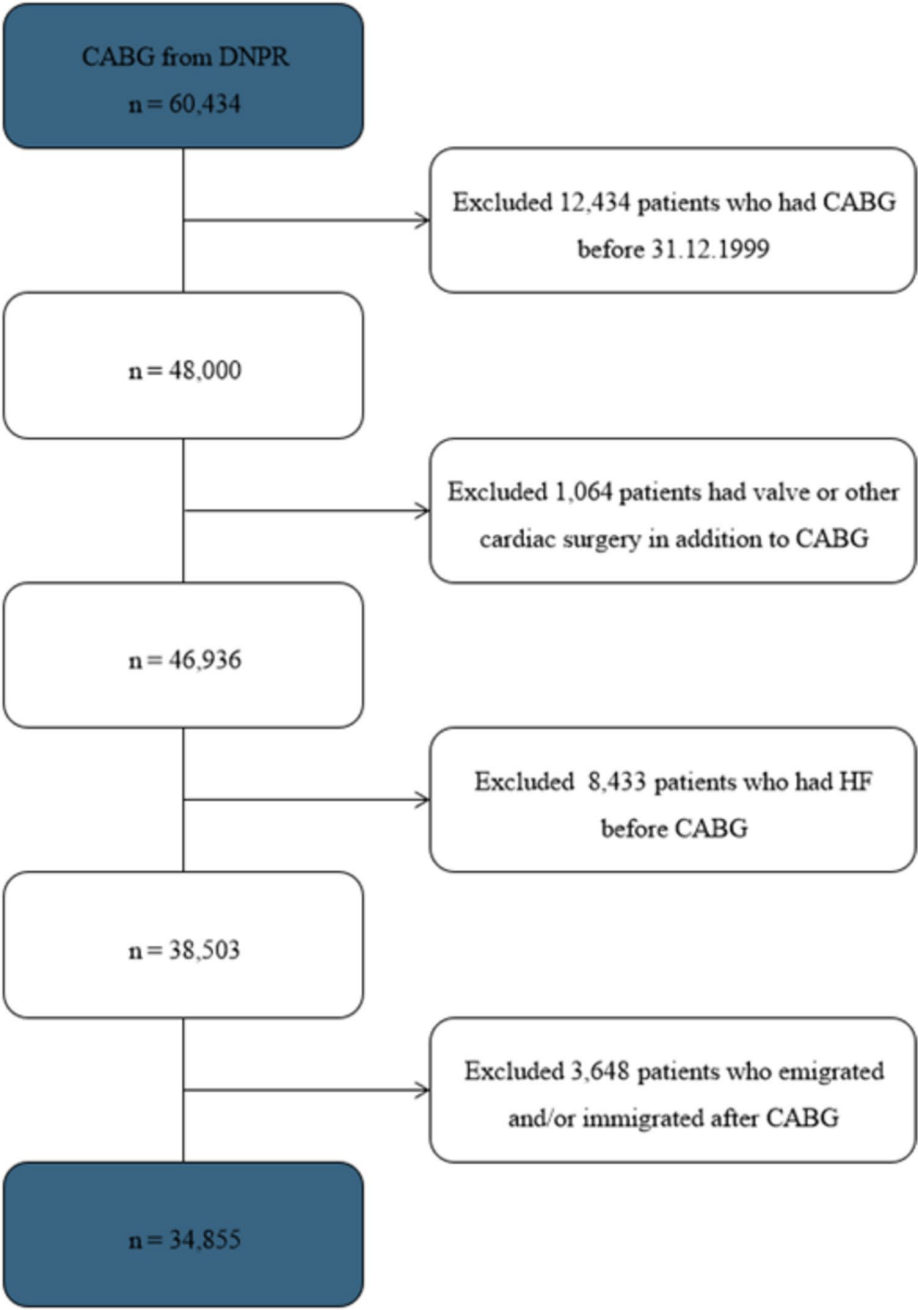
A flow diagram of the study population. A total of 60,434 patients were identified from the Danish National Patient Registry (DNPR). Following inclusion and exclusion criteria, 34,855 patients were included in this study. *CABG* Coronary artery bypass grafting, *HF* Heart failure, *DNPR* Danish National Patient Registry

A total of 34,855 patients were included in this study (Fig. 1), of whom 6909 (19.8%) had DM. The population was predominantly male (79.2%), and the mean age was 67.3 years (SD ± 9.4) (Table 1). Basic and high school education accounted for 42% of the total population each. The DM group presented with a greater frequency of all comorbidities and baseline medications, notably hypertension (67.8% versus. 47.5%), CKD (8.2% versus. 2.4%), ACE inhibitors (52.2% versus. 28.2%), and lipid modifying agents (83.0% versus. 69.6%) (Table 1). Furthermore, the DM group presented with a greater frequency of HF (30.3% versus. 24.3%) and death (28.1% versus. 26.0%). Subgroup analysis showed that of the non-DM, a total of 3,814 (13.6%) developed DM subsequent to their CABG. Among these732 developed HF.

**Table 1:** - Baseline Demographics and Clinical Characteristics in the Study Population

Variables	Total (*N* = 34,855)	DM group (*N* = 6909)	Non-DM group (*N* = 27,946)	*p* value
Sex, *n* (%)				0.014
Male	27,610 (79.2)	5389 (78.1)	22,212 (79.5)	
Mean age, *n* (sd)	67.3 (9.4)	67.1 (8.8)	67.3 (9.5)	0.037
Education, *n* (%)				< 0.001
0 – Basic school	14,323 (42.0)	3040 (44.7)	11,283 (41.3)	
1 – High school	14,315 (42.0)	2842 (41.8)	11,473 (42.0)	
2 – Medium education	4111 (12.1)	720 (10.6)	3391 (12.4)	
3 – High education	1360 (4.0)	196 (2.9)	1164 (4.3)	
Missing	746	111	635	
Comorbidities, *n* (%)				
Atrial fibrillation	4520 (13.0)	960 (13.9)	3560 (12.7)	0.011
Cancer	1774 (5.1)	337 (4.9)	1437 (5.1)	0.387
Chronic kidney disease	1239 (3.6)	566 (8.2)	673 (2.4)	< 0.001
Chronic obstructive pulmonary disease	2046 (5.9)	451 (6.5)	1595 (5.7)	0.010
Medication, *n* (%)				
Hypertension medication	17,964 (51.5)	4686 (67.8)	13,278 (47.5)	< 0.001
Acetylsalicylic acid	26,045 (74.7)	5564 (80.5)	20,481 (73.3)	< 0.001
Antiplatelet medication	6105 (17.5)	1410 (20.4)	4695 (16.8)	< 0.001
Non-vitamin K antagonist oral anticoagulant	465 (1.3)	115 (1.7)	350 (1.3)	0.009
Vitamin K antagonist	2010 (5.8)	466 (6.7)	1544 (5.5)	< 0.001
Lipid modifying agents	25,172 (72.2)	5732 (83.0)	19,440 (69.6)	< 0.001
Thyroid medication	1335 (3.8)	332 (4.8)	1003 (3.6)	< 0.001
ACE inhibitors	11,485 (33.0)	3606 (52.2)	7879 (28.2)	< 0.001
Diuretics	13,771 (39.5)	3696 (53.5)	10,075 (36.1)	< 0.001
Beta blockers	22,559 (64.7)	4507 (65.2)	18,052 (64.6)	0.327
Outcome, *n* (%)				
Heart failure	8878 (25.5)	2283 (30.3)	6785 (24.3)	< 0.001
Death	9130 (26.2)	2113 (28.1)	7252 (26.0)	< 0.001
Previous AMI	13,825 (39.7)	2882 (41.7)	10,943 (39.2)	< 0.001

Baseline demographics of the study population included sex, mean age, and education. Comorbidities and baseline medication were defined as dispensed prescription five years prior to coronary artery bypass grafting (CABG). Previous acute myocardial infarction (AMI) was defined as any AMI prior CABG. For further explanation concerning the ICD-10 and ATC codes used to define the comorbidities and medication, please refer to Online Resource Table 1

The prevalence of diabetes, hypertension, use of lipid modifying agents, atrial fibrillation, cancer, CKD, chronic obstructive pulmonary disease, and thyroid increased in the time intervals; years 2000–2005, 2005–2010, 2010–2015, and 2015–2020 (Online Resource Table 2).

The use of insulin among diabetes patients remained stable through the study period, the use of beta cell stimulating agent fell, while the use of Metformin, Glucagon-like peptide-1 analogs, Dipeptidyl peptidase-4 inhibitors, and Sodium Glucose Cotransporter 2 inhibitors rose (Online Resource Table 3).

Due to availability of laboratory data in the study period, baseline biochemistry was only available in 12–50% of the study population, and only from 2010 to 2020. Results are available in supplementary material (Online Resource Table 4).

### Risk of HF

**Fig. 2:**
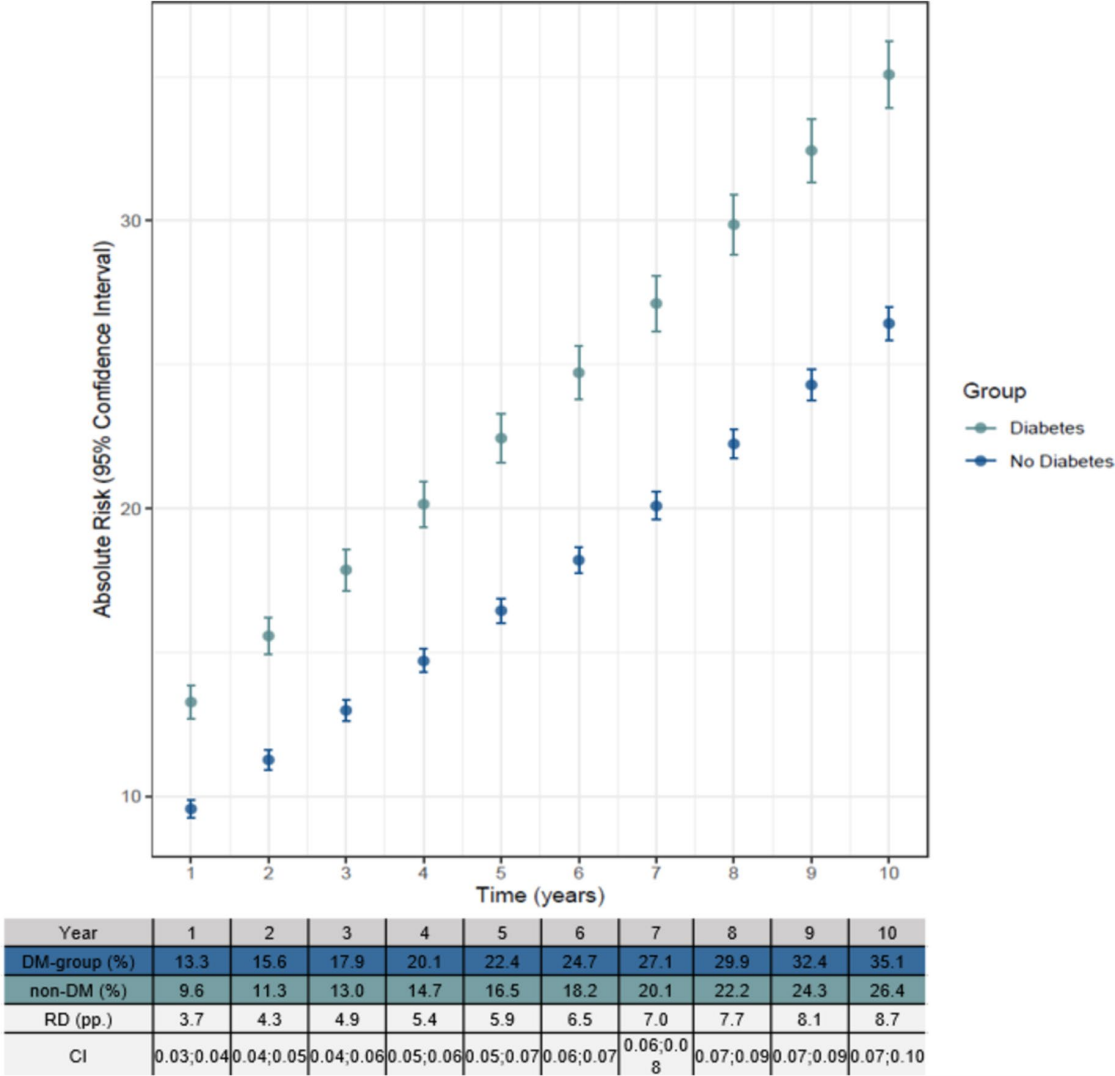
Visualization of ATE model showing the absolute risk (AR) of heart failure (HF) after a coronary artery bypass grafting (CABG). Each point shows the mean AR in the ten-year interval. The model is adjusted for sex, age, atrial fibrillation, cancer, chronic obstructive pulmonary disease, chronic kidney disease, hypertension, lipid modifying agents, and thyroid medication. The diabetes group includes both patients with type 1 diabetes and type 2 diabetes

**Fig. 3:**
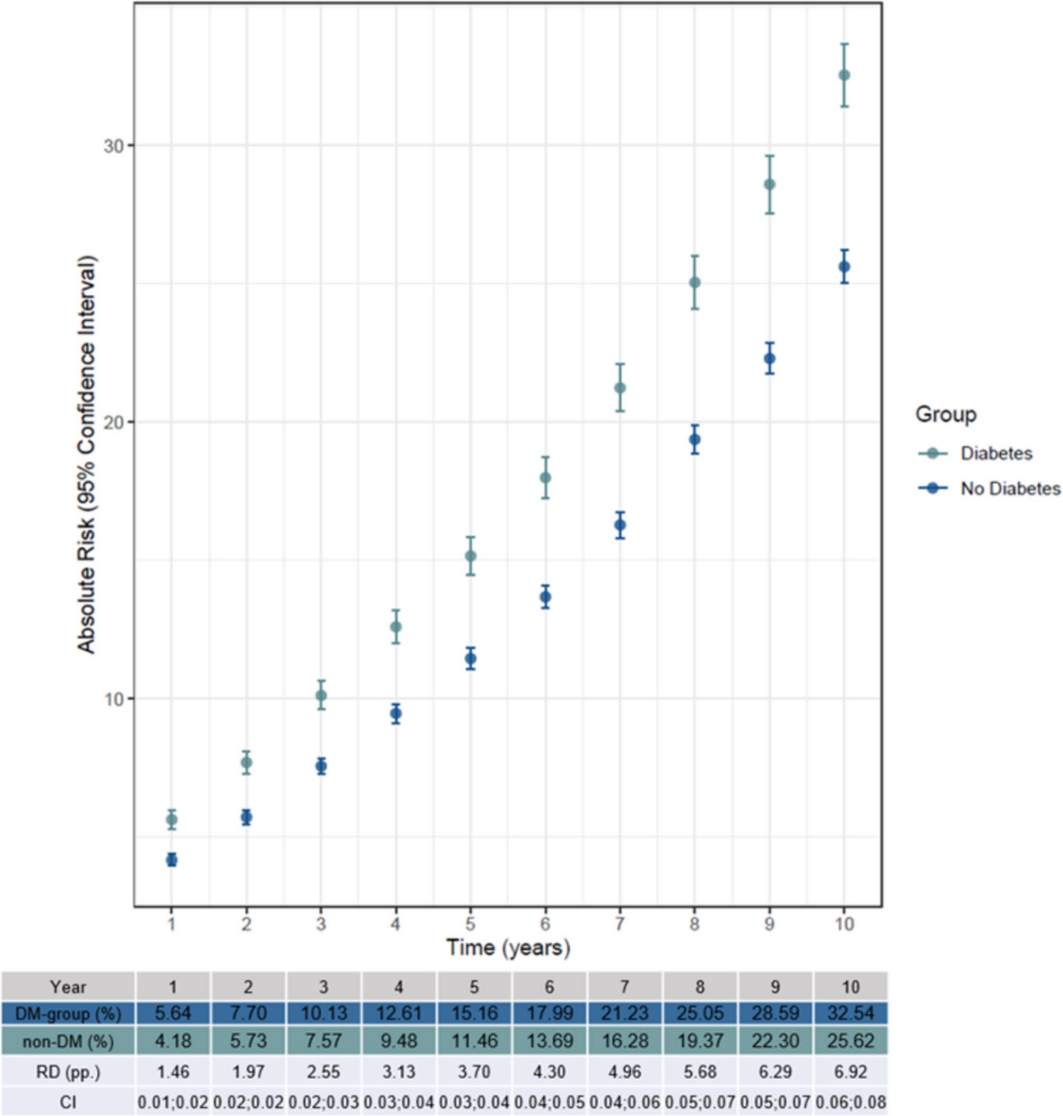
Visualization of ATE model showing the absolute risk (AR) of mortality after a coronary artery bypass grafting (CABG). Each point shows the mean AR in the ten-year interval. The model is adjusted for sex, age, atrial fibrillation, cancer, chronic obstructive pulmonary disease, chronic kidney disease, hypertension, lipid modifying agents, and thyroid medication. The diabetes group includes both patients with type 1 diabetes and type 2 diabetes

Models showed a significant difference in AR of HF (*p* < 0.001) after CABG between the DM and non-DM group with a greater AR in the DM group (Fig. 2). Furthermore, the hazard ratio for DM was 1.42 (*p* < 0.001) meaning diabetes is directly associated with increased risk of HF. The AR for the DM group was 13.3% for the 1st year and 35.1% for the 10th year. In the non-DM group, the AR was 9.6% for the 1st year and 26.4% for the 10th year. This reveals a higher AR in both groups in the ten years following CABG, however, showing a higher AR in the DM group for each year. The RD between the two groups increased each year ending with the RD being 8.7 pp. in the 10th year (Fig. 2). A stratified analysis reveals the AR of HF is greater for the DM group in the five-year intervals. Furthermore, the AR of HF is greater for both groups in year 2010–2015 compared to the other five-year intervals (Fig. 3).

### Risk of death

**Fig. 4:**
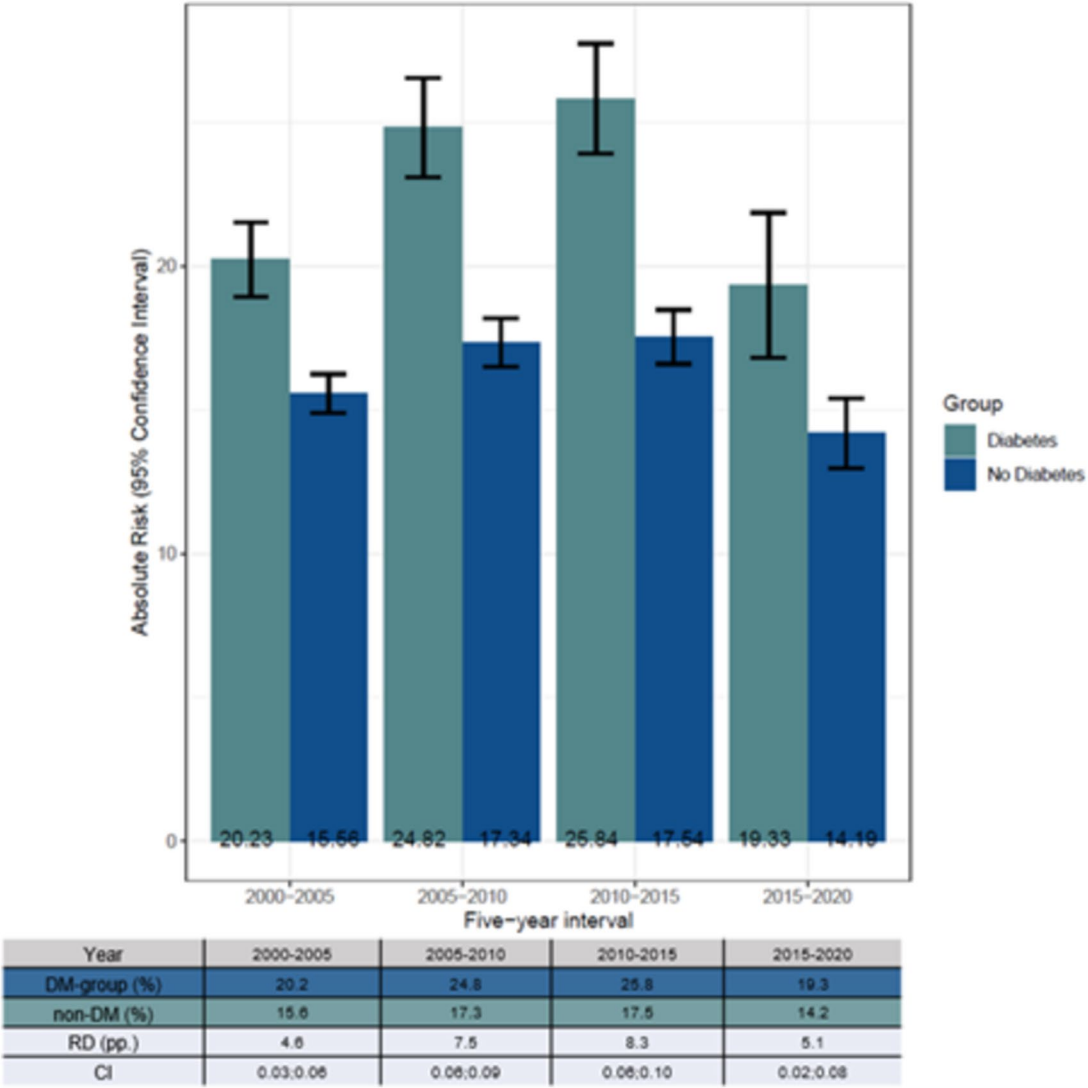
Stratified analyses showing the absolute risk (AR) of getting heart failure after a coronary artery bypass grafting (CABG) in four five-year intervals: 2000–2005, 2005–2010, 2010–2015, and 2015–2020. The model is adjusted for sex, age, atrial fibrillation, cancer, chronic obstructive pulmonary disease, chronic kidney disease, hypertension, lipid modifying agents, and thyroid medication

Analysis showed a significant difference in AR of death (*p* < 0.001) after CABG between the DM and non-DM group with a greater AR in the DM group (Fig. 4). The AR for the DM group was 5.6% for the 1st year and 32.5% for the 10th year. In the non-DM group, the AR was 4.2% the 1st year and 25.6% the 10th year. This means the AR for HF is higher than death in the 1st year, but toward year 10, the AR for HF and death are similar. The RD between the two groups increased each year ending with the RD being 6.9 pp. in the 10th year (Fig. 4).

### Additional analyses

Sensitivity analyses using AMI as a competing risk for HF together with having AMI and PCI as a secondary endpoint revealed no notable changes of results. Furthermore, adjusting for education level in the sensitivity analysis did not change the results. Excluding HF within 30 days postoperative did not materially change the results. Lastly, sensitivity analyses based on sex revealed no materially change in the results (Online Resource Fig. 1, Online Resource Fig. 2, Online Resource Fig. 3, Online Resource Fig. 4, and Online Resource Fig. 5).

## Discussion

This nationwide study based on all patients who underwent CABG from 2000 to 2020 found DM patients to be significantly more at risk of HF in a ten-year follow-up period. Furthermore, the AR continues to increase in the ten-year follow-up period and is not equalized between patients with DM and patient without DM (Graphical abstract).

With a substantial observational study population and extensive follow-up, this study concludes the great risk of HF being increased for people with DM, having no history of previous HF and/or AMI and receiving lipid modifying agents and/or hypertension medication, which is additionally consistent with previous minor studies [3, 9, 16]. Both the DM group and the non-DM group had a higher AR at the end of the ten-year period compared with the baseline, where the AR for the DM group increased from 13.3% to 35.1% and from 9.6% to 26.4% in the non-DM group. However, the AR is greater in the DM group, possibly because of further plaque formation, since CABG does not treat the main cause but decreases the ischemic effect of plaque formation in the affected coronary arteries. This, however, may not explain the risk in full, as we found a persisting increased risk of HF in a sensitivity analysis with acute myocardial infarction as an outcome in the HF risk model. As such, other factors including, comorbidities, including microvascular diseases, might develop, as well as nephropathy likely plays a role in the development of HF. One could speculate that the higher AR of AMI in patients with diabetes is among predisposing factors adding to the higher risk of HF in patients with DM, as suggested previously [29]. Despite this, analyses from the present study found a remaining significant risk difference, even after censoring patients who underwent revascularization or had an AMI. However, the exact causes of the high risk of HF in people with DM are still unclear, even though several risk factors could clarify the tendency, such as predispositions leading to a compromised myocardium as with advanced coronary atherosclerosis, stiffened myocardial tissue due to myocardial fibrosis, impaired calcium handling, deranged metabolism, and several other comorbidities all leading to systolic dysfunction and eventually HF [3, 9]. Furthermore, hypertension and impaired renal function are both considerable risk factors for developing HF [3]. In this study, 67.8% of the DM group were affected by hypertension compared to 47.5% of the non-DM group. In addition, 8.2% of the DM group were affected by CKD compared to 2.4% of the non-DM group, which might explain the high HF for the DM group as severe renal dysfunction is associated with a poor outcome after CABG [30]. Kainuma et al. investigated whether DM affects the outcome and survival after CABG in patients with left ventricular dysfunction and found a decline of the renal function in DM patients due to a steady decrease in the estimated glomerular filtration rate over time following CABG [12]. This implies impairment of the renal function is adverse, especially in DM patients with cardiac dysfunction, since it leads to poor kidney compensation, triggering a cycle leading to the risk of HF increasing significantly.

Aside from the risk factors mentioned previously, another possible risk factor of HF among DM patients could be the sodium retention. This is associated with high insulin levels due to insulin resistant states, and high levels of exogenous insulin delivery which contribute to blood volume overload in DM patients [9].

Even though we cannot delineate the underlying mechanism or factors behind the increased risk of HF, it is important as clinician and patient to be aware of the increased short- and long-term risk of HF following CABG and to consider HF as a cause when relevant symptoms including dyspnea arise.

### Strengths and limitations

This study used the Danish National Patient Registry which made it possible to extract extensive patient data. Previous studies with a similar aim included fewer patients in the total study population, compared to this study which included 34,855 in total, making the results more valid [9, 11, 12].

Inevitably, limitations were present due to the HF diagnosis being estimated at a sensitivity of 29% and a specificity of 99% [25]. Furthermore, new comorbidities and/or changes in medication throughout the study period were not included in this study, meaning the characteristics of the study population might have changed together with a wrongly interpretation of the results.

In this study, the DM group consisted of both T1DM and T2DM patients. Additionally, patients in this study population might have had undiagnosed T2DM, thereby being wrongly categorized in the non-DM group [5]. This is supported by Jørgensen et al. who estimated that 24% of all T2DM cases in Denmark were undiagnosed [31]. It is of interest to replicate this study, with divided DM groups (either T1DM versus T2DM, or non-insulin dependent DM versus insulin dependent DM), to see if it affects the results. Especially when considering that insulin dependent DM is associated with a poorer cardiac-specific survival rate compared to non-insulin dependent [8]. However, it is not possible to distinguish between T1DM and T2DM from the registry sources used in this study, but generally, people with DM are known to be of higher risk of IHD, rendering the results of this study as plausible.

The generalizability of the present study is limited by the fact that it only compares patients who have undergone CABG. The possibility of an overall lower risk of HF in patients with DM undergoing CABG, when compared with patients receiving percutaneous coronary intervention or optimal medical treatment, exists. A randomized study would be needed to fully clarify this.

## Conclusion

This Danish nationwide study demonstrates a significantly higher risk of HF up to ten years after CABG in patients with DM compared to patients without DM. Results were consistent in sensitivity analyses when (1) censoring for AMI, (2) adjusting for educational level, and (3) excluding HF in the first 30 days after CABG. Therefore, this study raises clinical awareness of a short- and long-term increased risk of HF in patients with DM compared to patients without DM.

## Supplementary Information

Below is the link to the electronic supplementary material.

Supplementary file1 (DOCX 522 KB)

## Abbreviations

CABG: Coronary Artery Bypass Grafting
HF: Heart Failure
IHD: Ischemic Heart Disease
CKD: Chronic Kidney Disease
AMI: Acute Myocardial Ischemia
AR: Absolute Risk
RD: Risk Difference
PCI: Percutaneous Coronary Intervention
T1DM: Type 1 Diabetes Mellitus
T2DM: Type 2 Diabetes Mellitus
