# Supplementary material for: Association between diabetes and heart failure after coronary artery bypass grafting: Danish register-based cohort study
Source: Clin Res Cardiol. 2025 Feb 24;114(5):640–50. doi: 10.1007/s00392-025-02594-8 (PMC12058951; doi:10.1007/s00392-025-02594-8)
Supplement: Supplementary file 1 — Supplementary file1 (DOCX 522 KB) [file 392_2025_2594_MOESM1_ESM.docx]

Association between diabetes and heart failure after coronary artery bypass grafting: Danish register-based cohort study

Clinical Research in Cardiology

Benedicte Bay Oxholm Brodersen^1^, Line Tribler Kristiansen^1^, Sidsel le Fevre Karlsen^1^, Jeppe Hauch^1^, Jan Jesper Andreasen^2,3^, Kristian H. Kragholm^3,4,5^, Maria Lukács Krogager^4^, Lars Valeur Køber^6^, Peter Christian Leutscher^7,8^, Dorte Melgaard^8,9^, Nisha I. Parikh^10^, Morten Schou^11^, Peter Søgaard^4^, Christian Torp-Pedersen^12^, Marc Meller Søndergaard^4^

^1^The Faculty of Medicine, Aalborg University, Aalborg, Denmark

^2^Department of Cardiothoracic Surgery, Aalborg University Hospital, Aalborg, Denmark

^3^Department of Clinical Medicine, Aalborg University, Denmark

^4^Department of Cardiology, Aalborg University Hospital, Aalborg, Denmark

^5^Unit of Clinical Biostatistics and Epidemiology, Aalborg University Hospital, Aalborg, Denmark

^6^Department of Cardiology, Rigshospitalet, Copenhagen University Hospital, Copenhagen

^7^Center for Clinical Research, North Denmark Regional Hospital, Hjørring, Denmark

^8^Department of Clinical Medicine, Copenhagen University, Denmark

^9^North Denmark Regional Hospital, Hjørring, Denmark

^10^Department of Medicine, Division of Cardiology, University of California San Francisco, San Francisco CA

^11^Department of Cardiology, Herlev and Gentofte Hospital, Copenhagen, Denmark

^12^Department of Cardiology, Nordsjællands Hospital, Copenhagen, Denmark

Corresponding author: Marc Meller Søndergaard^4^, [KJ01IH@dcm.aau.dk](mailto:KJ01IH@dcm.aau.dk)

# Supplemental tables

| **Online Resource Table 1: Supplemental material concerning ICD-10- and ATC codes** | | | |
| --- | --- | --- | --- |
| **Variable** | **ICD-10 code** | **ATC code** | **NPU-code** |
| **Definition of CABG** | **KFNA; KFNB; KFNC; KFND; KNFE** |  |  |
| Surgical anastomosis between a. mammaria interna and coronary artery | KFNA |  |  |
| Surgical anastomosis between a. gastroepiploica and coronary artery | KFNB |  |  |
| Coronary artery bypass grafting | KFNC |  |  |
| Coronary artery bypass grafting prosthetic | KFND |  |  |
| Coronary artery bypass grafting with use of free artery transplantation | KFNE |  |  |
| **Definition of DM** | **DE10; DE11; DE12; DE13; DE14; 250** | **A10**   - **Insulin: A10A** - **Betacell stumulating drugs: A10BB, A10BX02** - **Metformin: A10BA, A10BD07, A10BD08** - **DDP-4 inhibitors: A10BD08, A10BD07, A10BD19, A10BD13, A10BD21, A10BH01, A10BH02, A10BH03, A10BH04, A10BH05** - **SGLT2-inhibitors: A10BD, A10BK01, A10BK02, A10BK03** - **Glitazones: A10BG** - **GLP-1-inhibitors: B01BH, A10BJ, A10BX** |  |
| Type 1-DM | DE10 |  |  |
| Type 2-DM | DE11 |  |  |
| DM caused by malnutrition | DE12 |  |  |
| Other forms of DM | DE13 |  |  |
| Unspecified DM | DE14 |  |  |
| DM without mention of complication, type 2 or unspecified type, not stated as uncontrolled | 250 |  |  |
| **Definition of HF** | **DI50; DI420; DI428; DI429; DI110; DI130** |  |  |
| HF in general | DI50 |  |  |
| Cardiomyopathy | DI420; DI428; DI429 |  |  |
| Hypertensive heart disease | DI110; DI130 |  |  |
| Hypertensive kidney disease | DI120 |  |  |
| **Definition of AMI** | **I21** |  |  |
| **Definition of PCI** | **KFNG0** |  |  |
| **Comorbidities** |  |  |  |
| Hypertension | DI10; DI11; DI12; DI13; DI14; DI15; 40009; 40019; 40029; 40039; 40099; 40199; 40299; 40399; 40499 | Anti Adrenerg: C02A; C02B; C02C  Diuretic: C02DA; C03A; C03B; C03D; C03E; C03X; C02DA; C07B, C07C; C07D; C08G; C09BA; C09DA; C09XA52; C02L  Vasodilator: C02DB; C02DD; C02DG;  Beta Blocker: C07A; C07B; C07C; C07D; C07F  Calcium Antagonist: C07FB; C08; C08G; C09BB; C09DB  Renin-Angiotensin Inhibitor: C09AA; C09BA; C09BB; C09CA; C09DA; C09DB; C09XA02; C09XA52 |  |
| Chronic kidney disease | DN02 to DN08; DN11; DN12; DN14; DN18; DN19; DN26; DN158; DN159; DN160; DN162; DN163; DN164; DN168; DQ61; DE102; DE112; DE132; DE142; DI120; DM321B; DQ612; DQ613; DQ615; DQ619; 24902; 25002; 2558200; 58201; 58202; 58208; 58209; 58300; 583001; 583002; 583008; 583009; 58499; 59009; 59320; 75310; 75311; 75319; 79299; 40399; 40499; 44609; 44629 |  |  |
| Chronic obstructive pulmonary disease | DJ42; DJ43; DJ44; 49100; 49101; 49102; 49103; 49104; 49108; 49109; 49200; 49201; 49208; 49209 |  |  |
| Atrial fibrillation | DI48; 42793; 42794 |  |  |
| Cancer | DC00 to DC97 (excluding skin cancer DC44) and DC109 to DC140 |  |  |
| **Baseline medication** |  |  |  |
| Acetylsalicylic acid |  | B01AC06 |  |
| Antiplatelet medication (clopidogrel, prasugrel, ticagrelor) |  | B01AC04; B01AC22; B01AC24 |  |
| Anticoagulant medication (Non-vitamin K antagonist oral anticoagulant: rivaroxaban, apixaban, dabigatranetexilat, edoxaban and vitamin K antagonist: warfarin, phenprocoumon) |  | Non-vitamin K antagonist oral anticoagulant: B01AE07; B01AF01; B01AF02; B01AF03  Vitamin K antagonist: B01AA03; B01AA04 |  |
| Lipid modifying agents |  | C10 |  |
| Thyroid medication |  | H03 |  |
| ACE-inhibitors |  | C09AA |  |
| Diuretics |  | C02DA; C02L; C03A; C03B; C03D; C03E; C03X; C07B, C07C; C07D; C08G; C09BA; C09DA; C09XA52 |  |
| Beta blockers |  | C07A; C07B; C07C; C07D; C07F |  |
| **Baseline biochemistry** |  |  |  |
| eGFR |  |  | NPU28812, NPU28811, DNK35302 |
| Creatinin |  |  | NPU4998, NPU17559, NPU18016, NPU09101, NPU01807 |
| LDL-cholesterol |  |  | NPU01568, NPU10171, |
| HbA1c |  |  | NPU27300, NPU62912 |
| Glucose |  |  | NPU02192, DNK35842, NPU02187, NPU04092, NPU08972, NPU08509,  NPU10062-NPU10114 |

**Online Resource Table 1 - Supplemental material concerning ICD-10- and ATC codes**: Supplemental material concerning ICD-10- and ATC codes. Comorbidities and baseline medication were defined as dispensed prescription five years prior to CABG surgery [19]. The sensitivity and specificity of a heart failure diagnosis have previously been estimated to be 29% and 99%, respectively [25]

| Variable | Level | Years 2000-2005 (n=12,650) | Years 2005-2010 (n= 9,358) | Years 2010-2015 (n= 7,952) | Years 2015-2020 (n= 4,895) | Total (n=34,855) | p-value |
| --- | --- | --- | --- | --- | --- | --- | --- |
| Sex | Male | 9,882 (78.1) | 7,384 (78.9) | 6,321 (79.5) | 4,023 (82.2) | 27,610 (79.2) | <0.0001 |
| Age | mean (sd) | 66 (9.3) | 67.7 (9.4) | 68.4 (9.4) | 68 (9.2) | 67.3 (9.4) | <0.0001 |
| Education | 0 – Basic school | 5,763 (47.0) | 4,012 (43.6) | 3,014 (38.4) | 1,534 (31.9) | 14,323 (42.0) |  |
|  | 1 – High school | 4,862 (39.7) | 3,748 (40.8) | 3,430 (43.7) | 2,275 (47.4) | 14,315 (42.0) |  |
|  | 2 – Medium education | 1,242 (10.1) | 1,078 (11.7) | 1,045 (13.3) | 746 (15.5) | 4,111 (12.1) |  |
|  | 3 – High education | 390 (3.2) | 358 (3.9) | 364 (4.6) | 248 (5.2) | 1,360 (4.0) | <0.0001 |
|  | Missing | 393 | 162 | 99 | 92 | 746 |  |
| Diabetes | Yes | 2,149 (17.0) | 1,813 (19.4) | 1,788 (22.5) | 1,159 (23.7) | 6,909 (19.8) | <0.0001 |
| Hypertension medication | Yes | 5,418 (42.8) | 5,247 (56.1) | 4,721 (59.4) | 2,578 (52.7) | 17,964 (51.5) | <0.0001 |
| Lipid modifying agents | 1 | 7,609 (60.2) | 7,363 (78.7) | 6,358 (80.0) | 3,842 (78.5) | 25,172 (72.2) | <0.0001 |
| Thyroid medication | Yes | 454 (3.6) | 351 (3.8) | 343 (4.3) | 187 (3.8) | 1,335 (3.8) | 0.0657693 |
| Atrial fibrillation | 1 | 1,658 (13.1) | 1,174 (12.5) | 1,045 (13.1) | 643 (13.1) | 4,520 (13.0) | 0.5661489 |
| Cancer | 1 | 517 (4.1) | 449 (4.8) | 486 (6.1) | 322 (6.6) | 1,774 (5.1) | <0.0001 |
| Chronic kidney disease | 1 | 338 (2.7) | 324 (3.5) | 305 (3.8) | 272 (5.6) | 1,239 (3.6) | <0.0001 |
| Chronic obstructive pulmonary disease | 1 | 664 (5.2) | 597 (6.4) | 508 (6.4) | 277 (5.7) | 2,046 (5.9) | 0.0006 |

**Online Resource Table 2 – Baseline characteristics according to year of inclusion**

| **Variable** | **Level** | **Years 2000-2005 (n=2,149)** | **Years 2005-2010 (n=1,813)** | **Years 2010-2015 (n=1,788)** | **Years 2015-2020 (n=1,159)** | **Total (n=6,909)** | **p-value** |
| --- | --- | --- | --- | --- | --- | --- | --- |
| Insulin | Yes | 583 (27.1) | 523 (28.8) | 508 (28.4) | 341 (29.4) | 1,955 (28.3) | 0.4844 |
| Beta cell stimulating drugs | Yes | 986 (45.9) | 662 (36.5) | 363 (20.3) | 103 (8.9) | 2,114 (30.6) | <0.0001 |
| Metformin | Yes | 595 (27.7) | 848 (46.8) | 1,161 (64.9) | 763 (65.8) | 3,367 (48.7) | <0.0001 |
| GLP-1 analogs | Yes | 29 (1.3) | 31 (1.7) | 96 (5.4) | 106 (9.1) | 262 (3.8) | <0.0001 |
| DPP-4 inhibitor | Yes |  | 19 (1.0) | 168 (9.4) | 151 (13.0) | 338 (7.1) | <0.0001 |
| SGLT-2 inhibitors | Yes |  | 30 (1.7) | 84 (4.7) | 114 (9.8) | 228 (4.8) | <0.0001 |

**Online Resource Table 3 – Use of antidiabetic medication**

GLP-1: Glucagon-like peptide-1, DPP-4: Dipeptidyl peptidase-4, SGLT2: Sodium Glucose Cotransporter 2

| Variable | Level | No Diabetes (n=27,946) | Diabetes (n= 6,909) | Total (n=34,855) | p-value |
| --- | --- | --- | --- | --- | --- |
| HbA1c | Median [IQR] | 38 [35, 41] | 51 [44, 61] | 39 [36, 45] | <0.0001 |
|  | Missing | 23471 | 5071 | 28542 |  |
| Glucose | Median [IQR] | 5.9 [5.3, 6.8] | 9 [6.8, 11.9] | 6.2 [5.4, 7.7] | <0.0001 |
|  | Missing | 19725 | 4543 | 24268 |  |
| eGFR | <15 | 52 (1.6) | 28 (3.0) | 80 (1.9) |  |
|  | 16-30 | 62 (2.0) | 37 (3.9) | 99 (2.4) |  |
|  | 31-60 | 859 (27.0) | 278 (29.4) | 1137 (27.6) |  |
|  | 61-90 | 2067 (65.0) | 549 (58.1) | 2616 (63.4) |  |
|  | >90 | 138 (4.3) | 53 (5.6) | 191 (4.6) | <0.0001 |
|  | Missing | 24768 | 5964 | 30732 |  |
| LDL-cholesterol | Median [IQR] | 2.9 [2.1, 3.7] | 2.1 [1.6, 2.9] | 2.7 [2.0, 3.6] | <0.0001 |
|  | Missing | 19346 | 4388 | 23734 |  |
| Creatinine | Median [IQR] | 84 [71, 102] | 84 [67, 103] | 84 [70, 102] | 0.43 |
|  | Missing | 14820 | 3294 | 18114 |  |

**Online Resource Table 3 – Baseline biochemistry**

eGFR: Estimated glomerular filtration rate

**Supplemental figure**

**
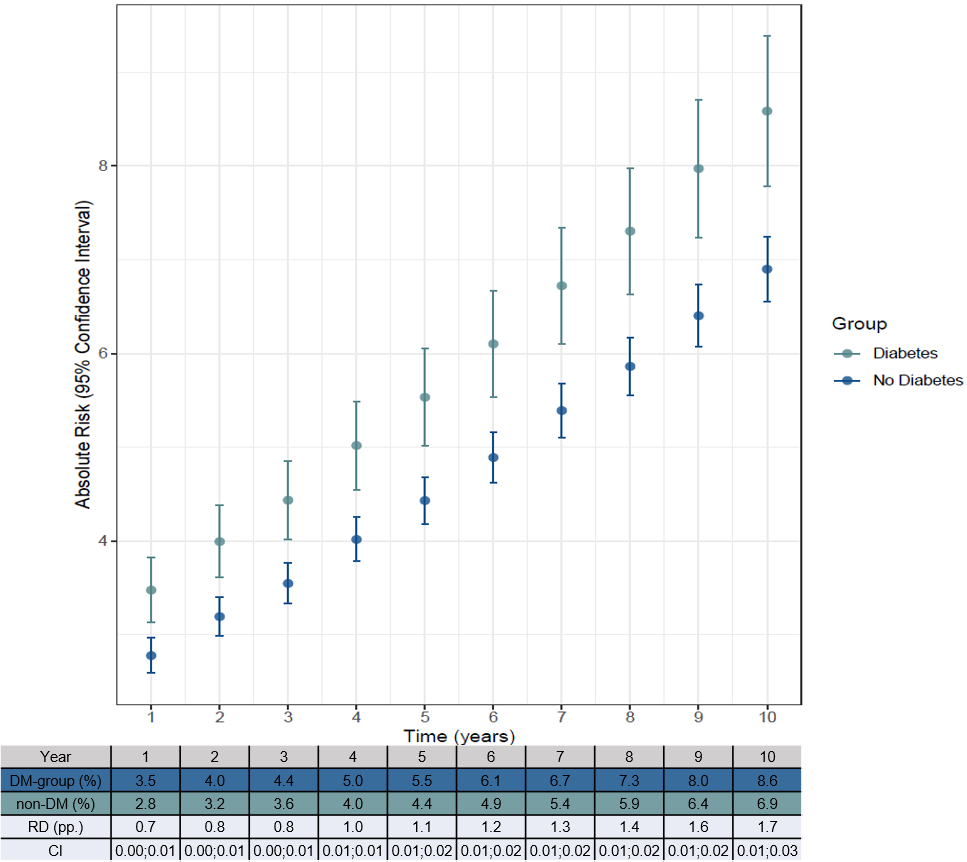
**

**Online Resource Fig. 1** Visualization of sensitivity analysis with acute myocardial infarction (AMI) as endpoint, showing the absolute risk (AR) of AMI after a coronary artery bypass grafting (CABG). Each point shows the mean-AR in the ten-year interval. The model is adjusted for sex, age, atrial fibrillation, cancer, chronic obstructive pulmonary disease, chronic kidney disease, hypertension, lipid modifying agents and thyroid medication. The diabetes group includes both patients with type 1 diabetes and type 2 diabetes

**
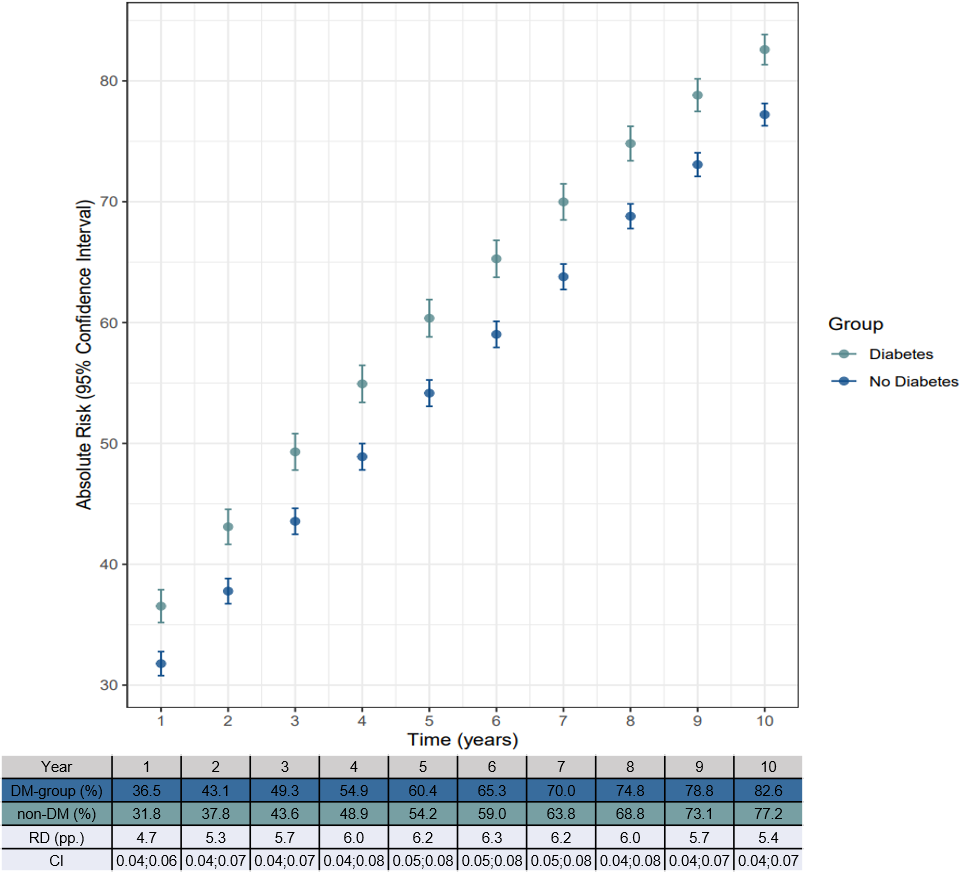
**

**Online Resource Fig. 2** Visualization of sensitivity analysis with acute myocardial infarction (AMI) and percutaneous coronary intervention as secondary endpoint, showing the absolute risk (AR) of HF after a coronary artery bypass grafting (CABG). Each point shows the mean-AR in the ten-year interval. The model is adjusted for sex, age, atrial fibrillation, cancer, chronic obstructive pulmonary disease, chronic kidney disease, hypertension, lipid modifying agents and thyroid medication. The diabetes group includes both patients with type 1 diabetes and type 2 diabetes


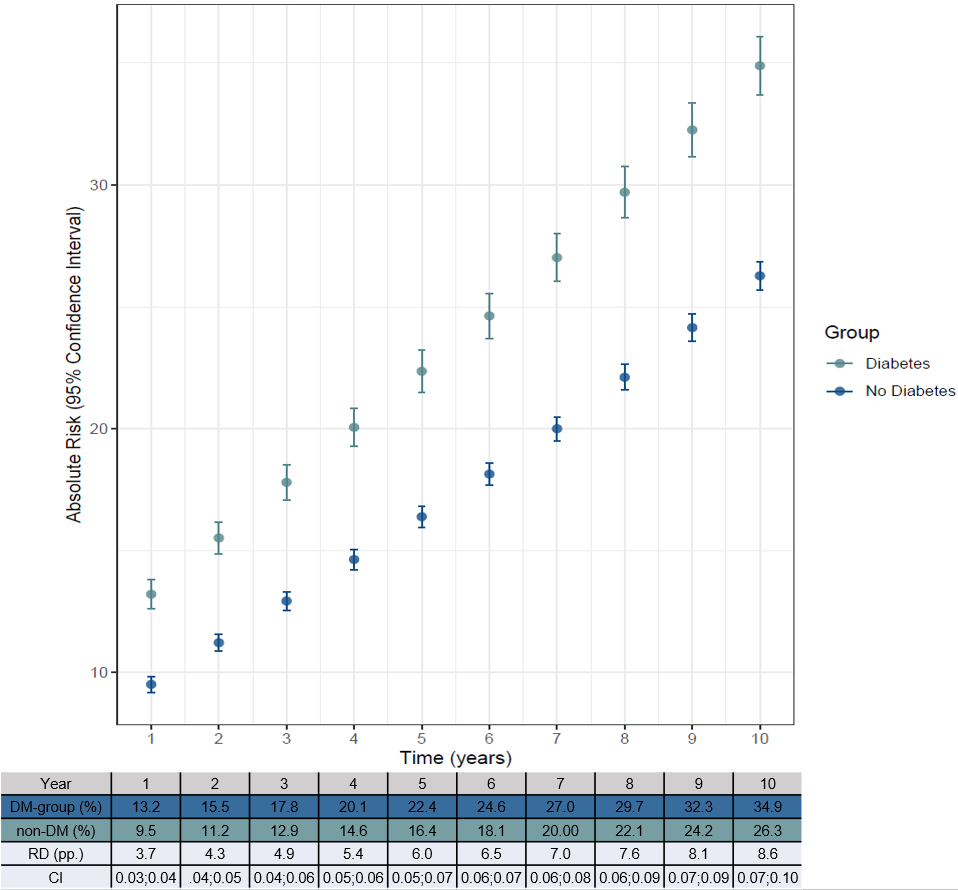


**Online Resource Fig. 3** Visualization of sensitivity analysis, showing the absolute risk (AR) of HF after a coronary artery bypass grafting (CABG). Each point shows the mean-AR in the ten-year interval. The model is adjusted for education, sex, age, atrial fibrillation, cancer, chronic obstructive pulmonary disease, chronic kidney disease, hypertension, lipid modifying agents and thyroid medication. The diabetes group includes both patients with type 1 diabetes and type 2 diabetes


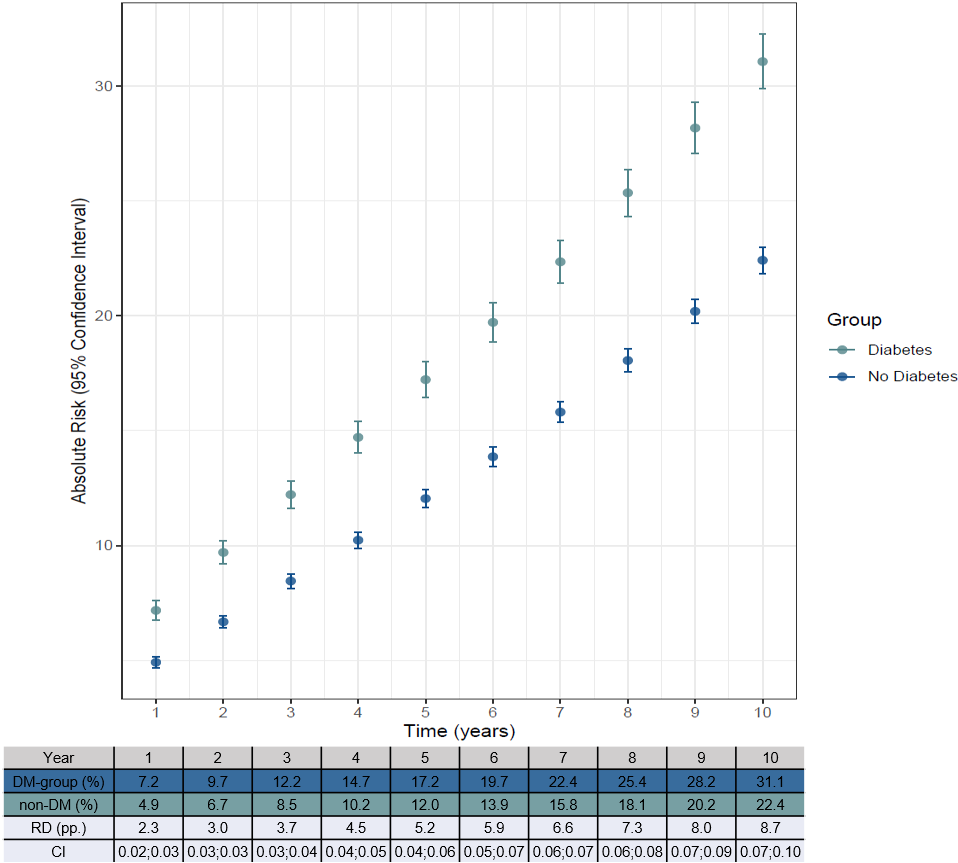


**Online Resource Fig. 4** Visualization of sensitivity analysis excluding patients who had heart failure (HF) within 30 days postoperatively, showing the absolute risk (AR) of HF after a coronary artery bypass grafting (CABG). Each point shows the mean-AR in the ten-year interval. The model is adjusted for sex, age, atrial fibrillation, cancer, chronic obstructive pulmonary disease, chronic kidney disease, hypertension, lipid modifying agents and thyroid medication. The diabetes group includes both patients with type 1 diabetes and type 2 diabetes

*
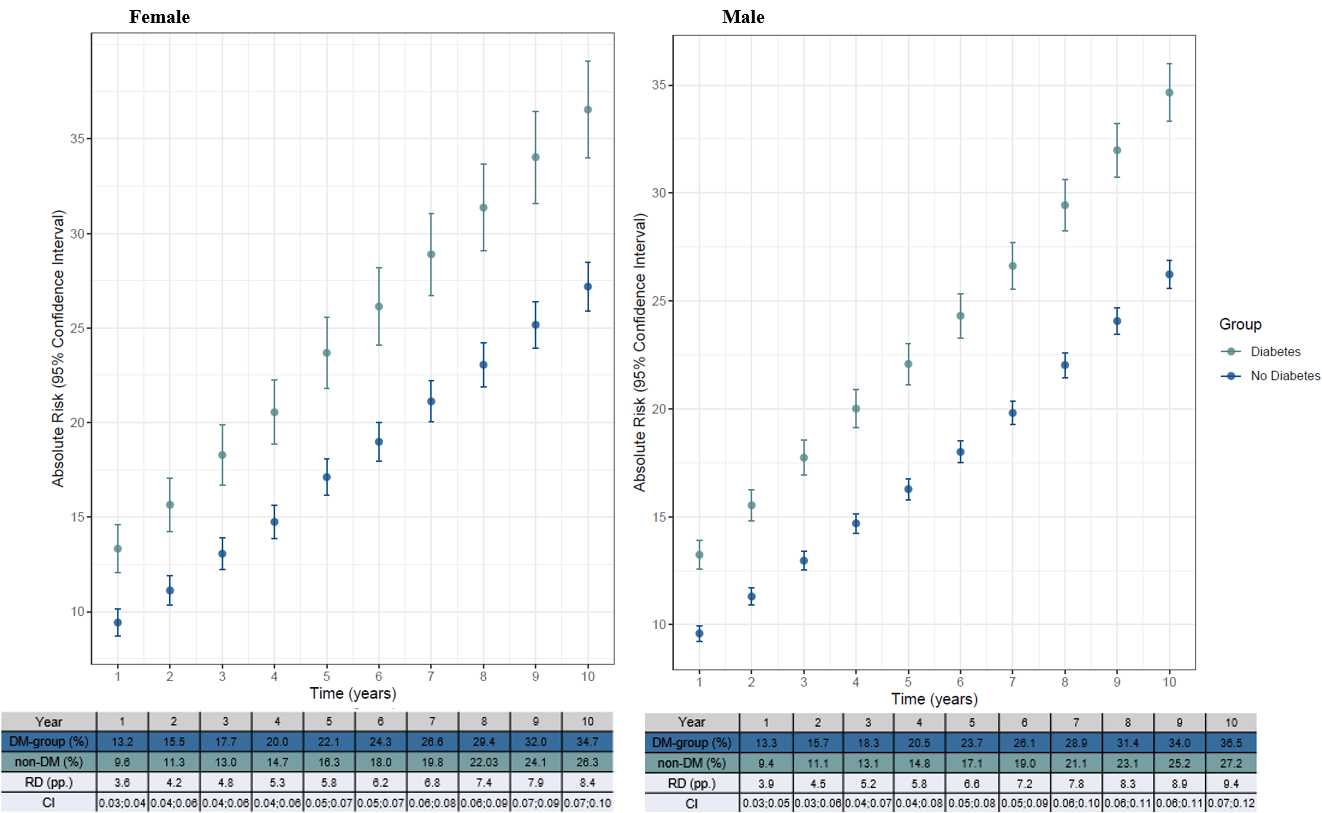
*

**Online Resource Fig. 5** Visualization of sensitivity analysis for each sex. Each point shows the mean-AR in the ten-year interval. The models are adjusted for age, atrial fibrillation, cancer, chronic obstructive pulmonary disease, chronic kidney disease, hypertension, lipid modifying agents and thyroid medication. The diabetes group includes both patients with type 1 diabetes and type 2 diabetes
